# Fabrication and Characterization of Ultra-High-Pressure (UHP)-Induced Whey Protein Isolate/κ-Carrageenan Composite Emulsion Gels for the Delivery of Curcumin

**DOI:** 10.3389/fnut.2022.839761

**Published:** 2022-02-25

**Authors:** Jiaqi Su, Linlin Wang, Wenxia Dong, Jiao Wei, Xi Liu, Jinxin Yan, Fazheng Ren, Fang Yuan, Pengjie Wang

**Affiliations:** ^1^Beijing Higher Institution Engineering Research Center of Animal Product, Key Laboratory of Precision Nutrition and Food Quality, Key Laboratory of Functional Dairy, Ministry of Education, College of Food Science and Nutritional Engineering, China Agricultural University, Beijing, China; ^2^College of Biological & Environmental Sciences, Zhejiang Wanli University, Ningbo, China; ^3^Department of Nutrition and Health, China Agricultural University, Beijing, China

**Keywords:** emulsion gel, ultra-high-pressure, whey protein isolate, κ-carrageenan, *in vitro* digestion, curcumin

## Abstract

The emulsion gels have attracted extensive interests due to their unique physical characters, remarkable stability, and control release properties of flavor and functional components compared to emulsions in liquid. In the current work, whey protein isolate (WPI)/κ-carrageenan (κ-CG) composite emulsion gels were fabricated based on the ultra-high-pressure (UHP) technology, in replacement of the traditional thermal, acid, or enzyme processing. Uniform composite emulsion gels could be fabricated by UHP above 400 MPa with minimum WPI and κ-CG concentrations of 8.0 and 1.0 wt%, respectively. The formation of UHP-induced emulsion gels is mostly attributed to the hydrophobic interaction and hydrogen bonding. The emulsion gels with different textures, rheology properties, and microstructures could be fabricated through adjusting the formulations (WPI concentration, κ-CG concentration, and oil phase fraction) as well as processing under different conditions (pressure and time). Afterward, curcumin-loaded emulsion gels were fabricated and subjected to an *in vitro* simulated gastrointestinal digestion in order to investigate the gastrointestinal fate of curcumin. *In vitro* simulated digestion results demonstrated that the UHP treatment significantly retarded the release of curcumin but had little impact on the bioaccessibility of curcumin. The results in this work provide useful information for the construction of emulsion gels through a non-thermal process, which showed great potential for the delivery of heat-sensitive bioactive components.

## Introduction

Generally, emulsions with a relatively low oil volume fraction are expected to exhibit liquid-like flow behavior. However, when a viscoelastic biopolymer solution or gel functions as a continuous phase, or an emulsion behaves as a viscoelastic material due to the structuring induced by inter-droplet attractions between the droplets or crowded arrangement of droplets, a complex colloidal material may therefore formed as both an emulsion and a gel. Such kind of soft-solid colloid material with a three-dimensional network structure is referred to as “emulsion gel” (1). As a typical semisolid system, the emulsion gels are able to exhibit the mechanical properties of both liquid and solid, as well as higher physical and oxidative stability compared with traditional emulsions. Moreover, the gel-like structure also enables the emulsions with better controlled properties, making them the ideal carriers for the encapsulation of hydrophobic bioactives.

The protein is one of the commonly used materials to construct food-grade emulsion gels. Protein-based emulsion gels can be classified into two types: (1) Emulsion-filled protein gel: Protein in the aqueous phase forms a polymer network in which oil droplets are uniformly dispersed, and the gel properties mainly depend on the nature of the protein matrix and (2) Protein-stabilized emulsion gel: Protein in the aqueous phase does not form a network structure but promotes the aggregation of droplets, while the gel properties mainly depend on the physical properties of the filled oil droplets. Actually, these two extreme structures typically coexist in a protein-based emulsion gel. That is, both the cross-linked protein molecules and the partially aggregated droplets are responsible for the formation of gel structures in these emulsion gels. To date, a variety of proteins were used to prepare emulsion gels, including whey protein (2), sodium caseinate (3), soy protein isolate (4), porcine serum protein (5), and so on.

As reported by abundant research studies, the protein-based emulsions can be converted into emulsion gels through several treatments (4–7), among which thermal treatment is the most commonly used one. However, the high temperatures (commonly higher than 65°C) would promote the degradation of thermally sensitive ingredients and result in the Maillard reaction within food if reducing sugar is involved, which have adverse effects on the quality, appearance, and flavor of food. Unlike heat-induced emulsion gels, cold-induced emulsion gels induced by acid or salt can be fabricated under mild conditions, and the process is more controllable, but the introduction of hydrogen and salt ions would also affect the microstructure and texture of gels, and further lead to the bad sensory characteristics of final food products, which limit the application of these gels in actual food systems.

Ultra-high-pressure (UHP) is a novel food processing technology that employs a pressure-transfer medium (such as water) to denature biological macromolecules and kill microbes under an extremely high pressure (100~1,000 MPa) (8). As a non-thermal processing technology, the UHP has been widely used in food processing and food sterilization due to its several advantages such as high efficiency, low consumption, safe operation, less pollution, and maximum retention of flavor substances in food (9). The UHP treatment under relatively low pressure can facilitate the unfolding of proteins, and the bridge flocculation would occur between the protein-coated oil droplets *via* non-covalent bonds. However, by further increasing the pressure, the unfolded monomers dissociated from the partial dimer would polymerize into more stable aggregates, the free sulfhydryl groups on the surface of oil droplets are liable to form disulfide bonds, thereby stabilizing the emulsions. In this case, a three-dimensional network structure can be constructed through non-covalent interactions between adjacent molecules, for instance, hydrogen bonding, disulfide bonding, Van der Waals force, and hydrophobic interaction force (10), enabling the encapsulation of water or other components. Besides, the formation of gel-network structure also contributes to improve the texture property, nutritional value, and functionality of food.

Due to its rich functional properties, such as emulsifying and gelling property, whey protein isolate (WPI) has been widely used in food industries. The WPI solution is liable to convert into gels, whose properties are dominated by multiple causal factors, e.g., protein composition, temperature, pH, ionic strength, etc. (11). Unfortunately, the gels fabricated from individual WPI exhibit low resistance against extreme environmental conditions, leading to a poor stability during storage as well as low bioavailability of encapsulated bioactive compounds. One of the promising methods to solve this problem is to incorporate polysaccharide within WPI gels, which is supposed to enhance the gel strength. Moreover, the complexation of protein and polysaccharide is beneficial to enhance of their functional properties, endowing them with improved interfacial properties and better protective effects on encapsulated sensitive compounds against processing and storage conditions. It is also capable of delivering bioactive compounds to specific gastrointestinal targets with optimal kinetics under mild conditions, achieving controlled release by chewing, pH changes, and enzymatic action. The previous research has confirmed that β-lactoglobulin and β-lactoglobulin-κ-carrageenan (CG) complexes can be induced into semisolid soft gels using UHP under a certain pressure treatment of 400 MPa (12). Chen et al. (13) have also reported that the UHP induction significantly improved the water holding capacity and increased the gel strength of chicken breast myosin/κ-CG composite emulsion gels with high pressure processing under 200 MPa.

To date, numerous studies have shown that complexation can occur between the amino groups on WPI and anionic polysaccharides by ion exchange (14–16). κ-CG is a polymeric hydrophilic anionic polysaccharide extracted from the cell wall of marine red algae, consisting of a repeating unit composed of the disaccharide, β-(1-3)-d-galactose-4-sulfate, and α-(1-4)-3,6-anhydro-d-galactose (17). The high sulfate content (around 15~40%) and an average relative molecular mass higher than 100 kDa endow κ-CG with a great potential in gelation (18). The previous research have reported that a cohesive κ-CG network can be fabricated in the presence of salt ions and cations such as K^+^, Ca2^+^, and NH^4+^, and the resulting spiral gels exert good controlled release properties of functional components. Verbeken et al. (19) believe that κ-CG is present in the composite gel in a manner that exists in the interstitial space of the protein network. The UHP can change the molecular structure and spatial conformation of polysaccharides, thereby affecting their physicochemical properties and forming gels (20–22). Therefore, it is theoretically feasible to construct WPI-κ-CG composite gels through UHP treatment.

Curcumin is a natural polyphenolic compound existed in the rhizome of the perennial herb curcuma longa (23). The health benefits of curcumin can be attributed to its various biological activities such as anti-inflammatory, anti-oxidant, anti-bacterial, anti-tumor activities, and so on (24). The potential health benefits of curcumin have generated a great interest in incorporating it into food, health-care products, and drugs. However, curcumin is extremely susceptible to oxidative deterioration due to the existence of hydroxy groups. Moreover, curcumin has also been shown to have poor solubility in aqueous phase, leading to a low compatibility to food matrix and reduced bioavailability (25). One of the effective methods to solve these problems is to encapsulate curcumin within delivery systems (26).

To date, the fabrication of emulsion gel is mostly limited to the induction by heat, enzyme, acid, and ion, and the research on the UHP-induced gel materials based on biological macromolecules concentrates mainly on the hydrogel, which partly stimulate the current work. The aims of this study were to prepare WPI and WPI/κ-CG emulsion gels by UHP induction and to find the effects of pressure, WPI, and κ-CG concentration. The mechanism of gel formation was also explored. The effects of UHP treatment conditions and gel composition on the *in vitro* digestive release rate of curcumin embedded in emulsion gels were studied for the first time, which provided evidence for the application of UHP-induced emulsion gels in the embedding and sustained release of functional ingredients.

## Materials and Methods

### Materials

The whey protein isolate was obtained from Davisco International, Inc, containing more than 95% protein. The κ-CG was acquired from CPKelco, Inc. Medium-chain fatty acid triglyceride (MCT) was purchased from Musim Mastika Oils and Fats. Curcumin (Cur, 98%) was purchased from the China National Medicine Group (Shanghai, China). Fluorescent dyes (Nile Red and Nile blue) were obtained from Sigma-Aldrich. All other chemicals used were of analytical grade.

### Preparation of Emulsion Gels

For WPI emulsion gels, a WPI solution (20 wt%) was prepared by dispersing 3.2 g WPI powder in 16 mL deionized water and stirring overnight to achieve complete dissolution. The WPI solution was then adjusted to pH 7.0 with 0.1M NaOH prior to mixing with 4 mL MCT oil under the shearing with an Ultra-Turrax (IKA, Germany) at a high speed of 10,000 r/min for 5 min. The resulting coarse emulsion was further homogenized three times using a Niro-Soavi Panda two-stage valve homogenizer (Parma, Italy) at 50 MPa. Afterward, a centrifuge tube containing 50 mL emulsion sample was placed in a nylon bag for vacuum sealing. The sample was immersed in the pressure-transfer medium water contained in the sample chamber of the UHP equipment. The boost rate and depressurization rate was set as 6.5 MPa/s and 20 MPa/s, respectively. The whole process took about 20 s ~ 2 min.

For WPI/κ-CG composite emulsion gels, an equal volume of WPI solution and κ-CG solution were mixed and stirred to swell overnight, then the mixed solution was treated on the basis of the method described above.

The WPI, κ-CG, and MCT concentrations, as well as the processing conditions (pressure and time) were summarized in Table 1.

**Table 1:** Composition and treatment of WPI/κ-CG composite emulsion gels.

**Sample ID**	**Composition**			**Treatment**	
	**WPI concentration (wt%)**	**κ-CG (wt%)**	**MCT fraction (v/v)**	**Processing pressure (MPa)**	**Pressure processing time (min)**
1	8	1	20	600	30
2	10				
3	12				
4	14				
5	16				
6	12	0.75			
7		0.6			
8		0.5			
9		0.4			
10				200	
11				400	
12	12	1		600	
13				600	20
14					10

### Rheology Analysis

The rheological properties of the samples were measured using a DHR-2 rheometer (TA Instruments, UK) at 25°C with a steel parallel plate coded as PP-40 (40 mm diameter, gap 1 mm). The emulsion gel samples were subjected to a dynamic frequency sweep test with the frequency oscillation varied from 0.1 to 100 rad/s at a 1% strain. All the dynamic tests were performed within the linear viscoelastic region. The elastic modulus (G′) and loss modulus (G″) were recorded.

### Texture Profile Analysis

The textures of samples were determined using a texture analyzer (TMS-Pro, FTC, America) operated under texture profile analysis (TPA) mode. The emulsion gel samples were taken out from centrifuge tubes and cut into cylinders (diameter 1.8 cm, height 2.0 cm) prior to placing in petri dishes. The samples were compressed to a distance of 1.0 cm with a P/6 probe at a speed of 100 mm·min^−1^. The interval between two consecutive compression cycles was 10 s. The hardness, springiness, chewiness, gumminess, and cohesiveness were calculated to characterize the textural properties of the samples. The TPA measurements were carried out for 5 independent replicates at 25°C.

### Fluorescence Spectroscopy

The Fluorimetric experiment was performed using an F-7000 fluorescence spectrophotometer (F-7000, Hitachi, Japan). The emulsion samples were diluted to 0.2 mg/mL (calculated on the basis of WPI) prior to placing in a quartz cell. The excitation wavelength was set as 290 nm, and the emission spectra were collected between 300 and 450 nm with a scanning speed of 100 nm·min^−1^. Both excitation and the emission slit widths were set at 5 nm. Each individual emission spectrum was the average of three runs.

### Circular Dichroism Spectroscopy

The Far-UV circular dichroism (CD) spectra of emulsion samples were recorded using a CD spectropolarimeter (Pistar π-180, Applied Photophysics Ltd. UK) in the far UV region ranged from 190 to 260 nm. The samples were diluted to 0.2 mg/mL (calculated on the basis of WPI) prior to placing in a quartz cell with a 1.0 mm optical path length. A constant nitrogen flush was applied during data acquisition. The secondary structure contents of the samples were estimated using Dichroweb: theonline Circular Dichroism Website http://dichroweb.cryst.bbk.ac.uk (27).

### Fourier Transform Infrared Spectroscopy

The Fourier transform infrared spectroscopy (FTIR) was employed to evaluate the vibration of functional groups of emulsion gel samples. Briefly, 2.0 mg lyophilized sample was mixed with 198 mg pure potassium bromide (KBr) powder. The mixture was grounded into fine powder and pressed into a transparent thin slice. Afterward, the samples were subjected to FTIR analysis using a Spectrum 100 Fourier transform infrared spectrometer (PerkinElmer, UK). All the samples were scanned from 400 to 4,000 cm^−1^, and scanning was performed 16 times with a resolution of 4 cm^−1^. Pure KBr slice was measured as a baseline.

### Microstructure Observation

The microstructure of emulsion gel samples was observed using a field emission scanning electron microscope (SEM, SU8010, Hitachi) and a confocal laser scanning microscopy (CLSM) (Leica TCS SP5, Leica MicrosystemsInc., Heidelberg, Germany).

For SEM observation, the lyophilized samples were adhered onto a specimen and sputter-coated with a gold layer to avoid charging under the electron beam. The samples were examined at an acceleration voltage of 3.0 kV using a JSM-6701F instrument.

For CLSM observation, the two dyes, Nile Blue (0.2 wt%) and Nile Red (0.2 wt%), were dissolved in absolute ethanol and sonicated for 5 min to ensure the complete dissolution, applying as dyes of WPI and MCT, respectively. A mixed staining solution prepared by mixing an equal volume of Nile Blue and Nile Red dye solution was used to dye the emulsion samples. The dyed samples were subsequently placed in the groove of concave microscope slides and gently covered with a cover slip prior to observe with a 100-fold oil mirror. A Helium–Neon (He–Ne) laser excitation source was excited at 633 nm for the Nile blue, whereas an argon laser excitation source was excited at 488 for the Nile red. The combined pictures originated from two channels were acquired through ZEN Imaging Software.

### Determination of Molecular Force

A WPI solution (20 wt%) and a κ-CG solution (2 wt%) were prepared with NaCl, urea, and propylene glycol solutions at concentrations of 0.4, 0.8, 1.2, 1.6, and 2.0 mol/L, respectively. Afterward, the above-mentioned solutions were employed to prepare emulsion samples with the method described in section Preparation of Emulsion Gels before subjecting to a UHP-treatment at 600 MPa for 30 min to form emulsion gels. The molecular structure of emulsion gel samples was determined by measuring the texture properties of each sample with a TPA analysis as described in section Texture Profile Analysis (TPA).

### Preparation of Curcumin-Loaded Emulsion Gels

An oil phase was formed by dissolving 1.0 g curcumin powder in 200 mL of MCT oil prior to ultrasonic-treating for 20 min with a JY32-11N Ultrasonic homogenizer (Ningbo Scientz Biotechnology Co. Ltd, China) to achieve complete dissolution. The curcumin-loaded emulsion gels were fabricated with the same method described in section Preparation of Emulsion Gels.

### *In vitro* Digestion Behavior

#### Simulation Gastrointestinal Digestion

A simulated gastrointestinal tract (GIT) model was used to determine the potential gastrointestinal fate of emulsion gels. The results of our preexperiments have shown that the simulated mouth digestion had almost no effect on the digestion of emulsion gels; the simulated mouth digestion phase was not involved in the following experiments. However, the emulsion gels were broken into small fragments using a MJ-BL25B2 blender before the experiment to better simulate the initial state of samples in stomach.

Gastric phase: 1.0 g of broken emulsion gel samples were mixed with 10 ml of simulated gastric fluid (SGF), which contained NaCl (2 mg/mL), HCl (4 mL/L), and pepsin (3.2 mg/mL). Then, the pH of the mixtures was adjusted to pH 1.5 and stirred at 37°C for 60 min at 100 rpm.

Small intestine phase: 20 mL of gastric digestion juice were adjusted to pH 7.0 prior to mixing with an equal volume of simulated intestinal fluid (SIF) containing K_2_HPO_4_ (6.8 mg/ml), NaCl (8.775 mg/ml), bile salts (10 mg/ml), and pancreatin (3.2 mg/ml). Then, the pH of the mixture was adjusted to pH 7.0 and continuously shook (100 rpm) at 37°C for 120 min.

#### Droplet Size Determination

To explore the changes in the droplet size and size distribution of emulsion gels after SGF and SIF digestion, the SGF and SIF digestion juices were measured using a Zetasizer Nano-ZS90 (Malvern Instruments, Worcestershire, UK) based on the principle of dynamic light scattering (DLS).

#### Release of Curcumin

The release of curcumin was characterized after SIF digestion according to the method described in a previous work. An aliquot of SIF digestion juice was subjected to centrifugation with a CL10 centrifuge (Thermo Scientific, Pittsburgh, PA, USA) at 16,000 rpm for 30 min at 4°C. The supernatant containing solubilized curcumin was collected, on behalf of the “micelle” fraction. The micelle solution was then mixed with an equal volume of ethanol, vortexed, and centrifuged at 4,000 rpm for 10 min at 25°C. The top layer was then collected, whereas the bottom layer was subjected to the above-mentioned process again to ensure the complete extraction of curcumin. The digestive fluid was regularly sampled with an interval of 30 min. The concentration of curcumin extracted from the initial emulsion and micelle fraction was measured using a UV–visible spectrophotometer (Shimadzu, Model UV 1800; Japan) at 446 nm. The release rate (%) was calculated with the following formula:


$$\begin{array}{c} Release\;rate\;\left(\%\right)=\frac{C_{1}}{C_{0}}\times 100 \end{array}$$

Where C_1_ and C_0_ were the content of curcumin in the micelle fraction and initial emulsion gel samples, respectively.

### Statistical Analysis

The samples were prepared in duplicate, and the measurements were performed in triplicate. The data were analyzed using the software package statistical product and service solutions (SPSS) 18.0 (SPSSInc., Chicago, USA). The results were reported as the mean value and SD of two separate injections. The statistical differences were determined by one-way ANOVA with Duncan procedure, and the differences of main effects were identified to be significant with *p* < 0.05.

## Results and Discussions

### Apparent Characteristics of Emulsion Gels With Different Processing Conditions

**Figure 1:**
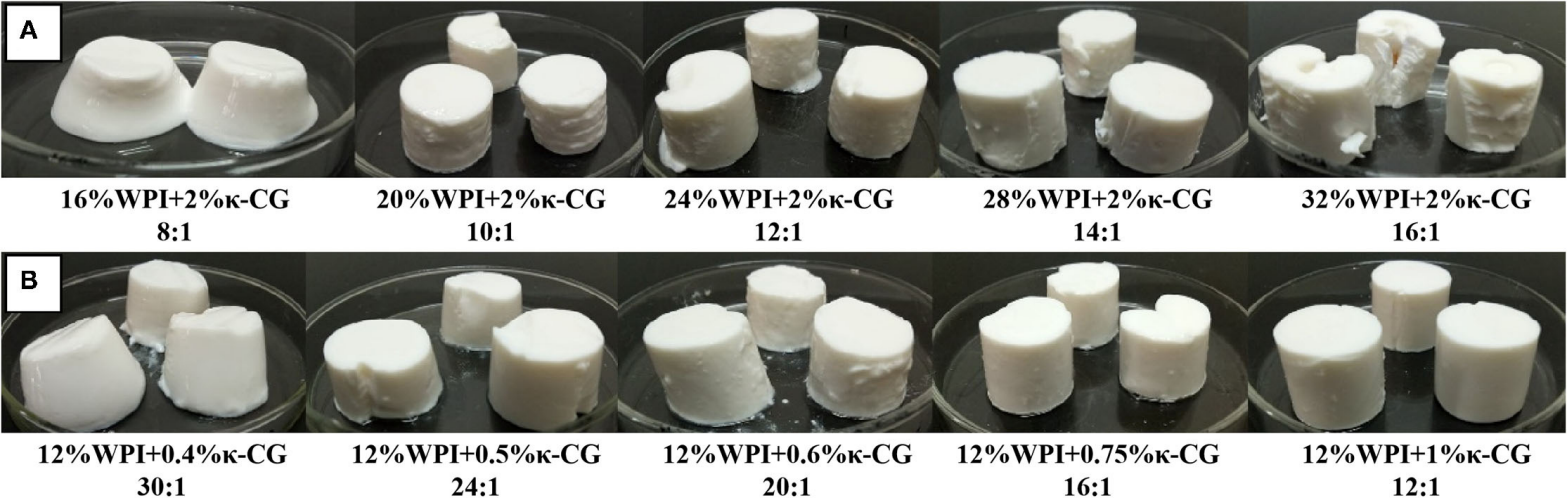
Visual appearance of WPI emulsion gels **(A)** and WPI/κ-CG composite emulsion gels **(B)** composed of different components.

The WPI/κ-CG composite emulsion gels with a WPI concentration lower than 12% were found with water leakage phenomenon, as shown in Table 2. It could be concluded that 12% was the minimum concentration for the formation of homogenized emulsion gels, and therefore was chosen to fabricate emulsion gels with different WPI/κ-CG ratios. The appearance photographs of WPI and WPI/κ-CG composite emulsion gel samples with different processing conditions were shown in Figure 1. Unlike a WPI emulsion, the WPI/κ-CG composite emulsions could be constructed into emulsion gels with an extremely low WPI level under UHP treatment. However, the WPI/κ-CG composite emulsion gel formed by a critical WPI concentration (12%) processed at 600 MPa for 30 min was more soft and viscous compared with the emulsion gels formed by WPI emulsions with an equal WPI concentration. Besides, the WPI/κ-CG composite gels with relatively low concentration of WPI and κ-CG were moister, accompanied by the occurrence of a water leakage phenomenon.

**Table 2:** The physical state of WPI/κ-CG composite emulsion gels with different formulations.

**WPI concentration (wt%)**	**κ-CG concentration (wt%)**	**Mass ratios between WPI and κ-CG**	**Texture and shape**
8	1	8:1	Soft, sticky, collapse after placement, water seepage
10	1	10:1	Soft, sticky, poor elasticity, lightly seepage after placement
12	1	12:1	Soft, poor elasticity, brittle
14	1	14:1	Relatively hard, deform after extrusion, rough surface, fragile
16	1	16:1	Hard, deform after extrusion, rough surface, fragile
12	0.75	16:1	Soft and sticky, poor elasticity, water seepage after placement
12	0.6	20:1	Very soft and sticky, poorly collapsed after placement, more water seepage
12	0.5	24:1	Very soft and sticky, poor elasticity, obvious collapse after placement, more water seepage
12	0.4	30:1	Very soft and sticky, collapses immediately after placement, obvious fluidity

Overall, the WPI/κ-CG composite emulsion gels were more brittle with a higher mobility, and easily destroyed (different degrees of collapses occurred after standing for 10 min). However, the introduction of κ-CG contributed to increase the hardness and decrease the mobility of the WPI emulsion gels, as well as mitigate the water leakage phenomenon. For example, the emulsion gels formed with 10% (w/v) WPI and 1% (w/v) κ-CG were deformed after extrusion when the oil fraction was 30% (w/w), but were cracked or even broken when the oil fraction exceeded 40% (w/w). The high brittleness of WPI/κ-CG composite gels indicated that κ-CG can promote the formation of a fine strand network, which endowed the gel with a decreased stress and an increased fracture strain (20). In summary, the addition of κ-CG could have a positive impact on the formation of gel structure in WPI emulsions.

### Rheological Properties of Emulsion Gels

**Figure 2:**
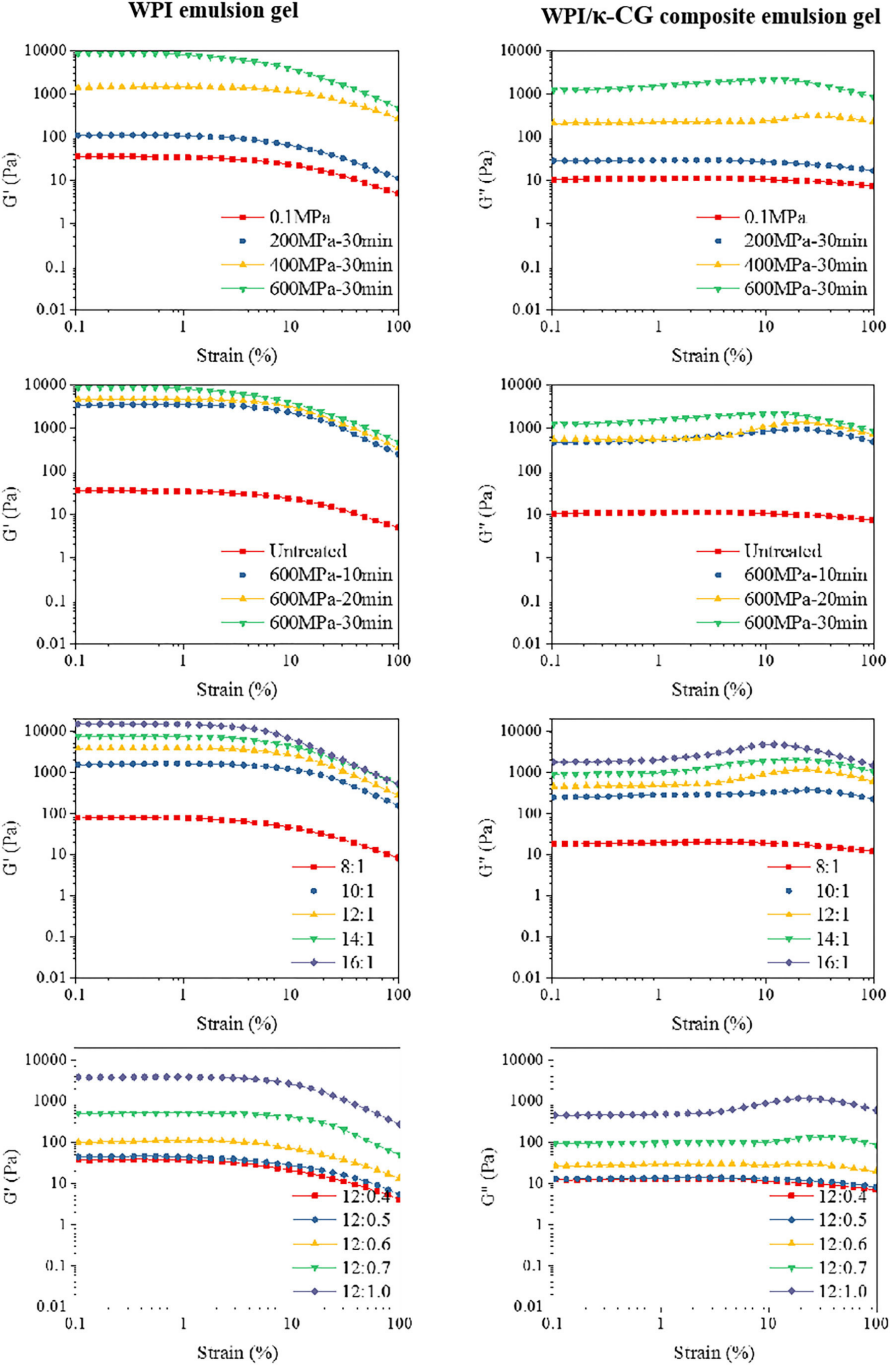
Strain scan curves of WPI and WPI/κ-CG composite emulsion gels [The concentration ratio number “a:b” in the figure represents “WPI%(w/v):κ-CG%(w/v)” in aqueous phase.].

In this experiment, the storage modulus G' (elastic modulus) and the loss modulus G” (viscous modulus) were determined to characterize the rheological properties of the WPI and WPI/κ-CG emulsion gels. G' is a measure of the energy stored and subsequently released in each deformation cycle, which is on behalf of the elastic properties of the samples, and G” is a measure of the amount of heat dissipation, representing the viscous properties of the samples. As shown in Figure 2, the dynamic strain sweep tests were conducted on the emulsion gel samples. Considering the fact that all the samples were determined with a linear viscoelastic region between 1 and 10% strain, 1% strain was selected as a fixed strain value in the frequency sweep tests. The critical strain value (γ_c_) was decreased as the treatment pressure and time increased, as well as the WPI and κ-CG concentration. At the same time, the strain-amplitude dependence type of emulsion gels changed from a strain thinning type (G' and G” decreasing) to a weak strain overshoot type (G' decreasing, G” increasing followed by decreasing), indicating that the structure in emulsion gels was changed and the stress that the gel structure can withstand was reduced (28).

The dynamic frequency sweep test was conducted to evaluate the frequency dependence of the G' and G' of emulsion gel samples with different compositions and processing conditions. As shown in Figure 2, the moduli were found to be in a frequency-dependent manner. It could be also observed that G' was higher than G” in all the emulsion gel samples, revealing that these samples behaved between true gels and weak gels (29, 30). Besides, the G' was highly closed to G' for samples with relatively low WPI and κ-CG concentrations. However, the difference between G' and G” was increased as the increasing WPI and κ-CG concentration as well as treatment pressure, which indicated that the emulsion samples further converted into true gels (31).

Table 3 shows the fitting result of the storage modulus and power-law model to describe the angular frequency (ω) dependence on the modulus. The G' *versus* ω of the samples fitted well to the power law model after the UHP treatment, with a pressure higher than 400 MPa. As the treatment pressure and time were increased, the K' and K'/n' was increased, suggesting that the UHP treatment promoted the formation of non-covalent bonds and enhanced the gel strength of the samples (32, 33). When the WPI concentration was 12% (w/v), the modulus of the samples showed a better imitative effect with a κ-CG concentration higher than 0.5% (w/v). For the samples with better gel properties, the fitting parameter n' for power law model was decreased as the WPI and κ-CG concentration increase, accompanied by a significantly increased K' value. This was consistent with the report of Elsevier (34), who had proposed that the rheological behavior of emulsions can be regulated by the protein/polysaccharide ratio, and a higher protein/polysaccharide ratio is beneficial for the interactions between protein molecules.

**Table 3:** Power Law parameters for WPI/κ-CG composite emulsion gel of different processing conditions and composition ratios.

**Processing conditions**	**WPI concentrations (wt%)**	**κ-CG concentrations (wt%)**	**G'= K'•ω^n′^**			
			**K'**	**n'**	**R^**2**^**	**K'/n'**
—	12	1	17.95288 ± 1.60542	0.32318 ± 0.02539	0.87605	55.55071
200 MPa−30 min			59.67279 ± 2.26976	0.11821 ± 0.01331	0.75812	504.8032
400 MPa−30 min			993.69014 ± 12.08667	0.11641 ± 0.00427	0.96466	8536.124
600 MPa−10 min			2109.82495 ± 19.75605	0.11257 ± 0.0033	0.97716	18742.34
600 MPa−20 min			3710.52839 ± 30.47441	0.08493 ± 0.00302	0.96614	43689.25
600 MPa−30 min			5364.19299 ± 65.84353	0.10253 ± 0.00439	0.95248	52318.28
	8		42.16605 ± 2.42844	0.25085 ± 0.01731	0.89503	168.0927
	10		834.01794 ± 8.61492	0.13879 ± 0.00351	0.98347	6009.208
	14		5795.58181 ± 109.61936	0.08898 ± 0.00691	0.85814	65133.53
	16		11001.57157 ± 63.66716	0.0879 ± 0.00212	0.98427	125160.1
	12	0.4	18.81607 ± 1.27015	0.12802 ± 0.0233	0.56204	146.9776
		0.5	4.9251 ± 1.55024	0.81397 ± 0.0759	0.88812	6.050714
		0.6	47.86639 ± 3.908	0.31371 ± 0.02334	0.88784	152.5817
		0.7	337.98244 ± 7.21355	0.15368 ± 0.00712	0.94709	2199.261

### Texture Analysis of Emulsion Gels

**Figure 3:**
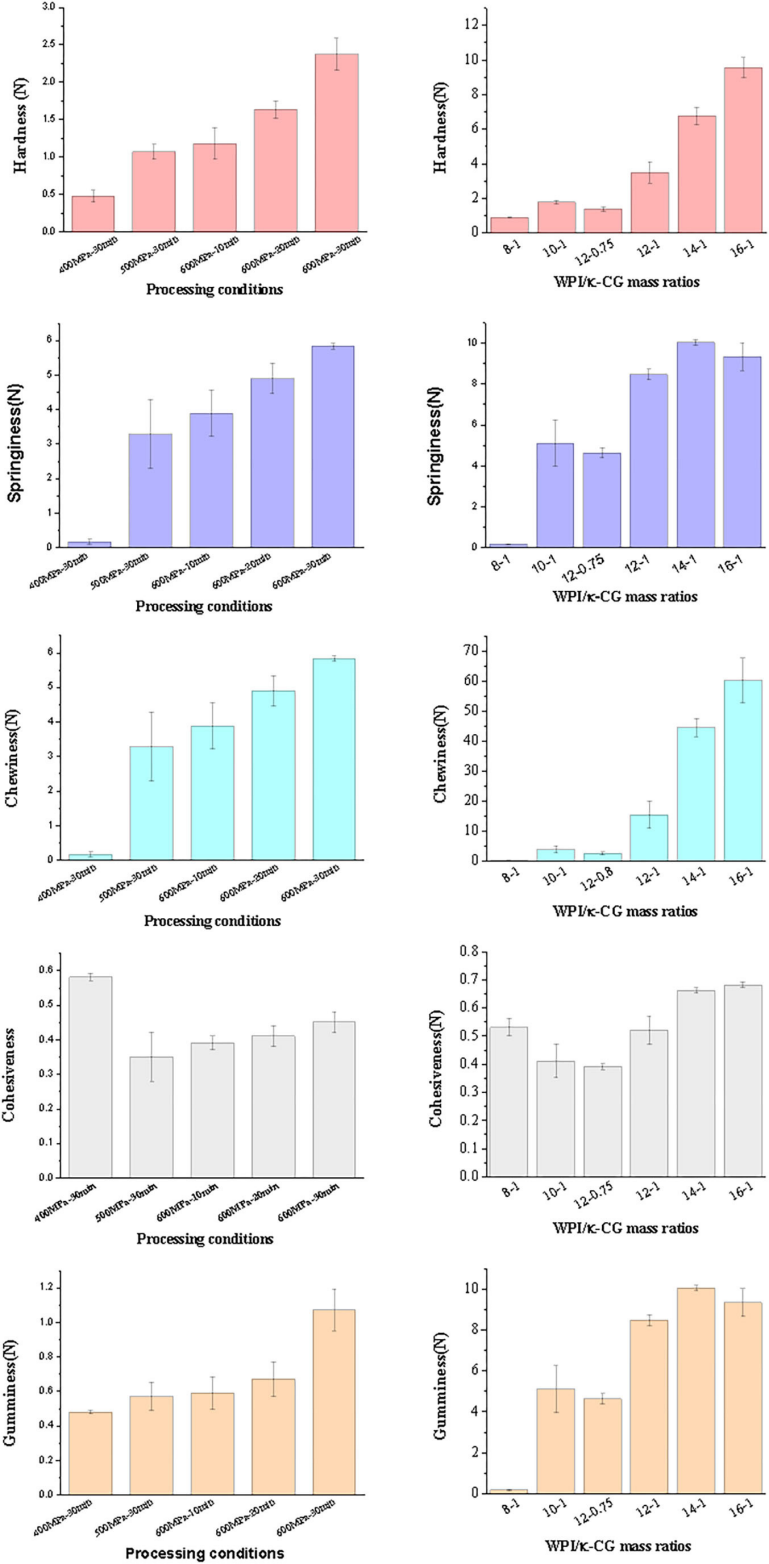
Texture of emulsion gels of different processing conditions and composition ratios [The concentration ratio number “a:b” in the figure represents “WPI%(w/v):κ-CG%(w/v)” in aqueous phase.].

The texture properties of emulsion gel samples were determined and the results are shown in Figure 3. Obviously, the hardness, elasticity, chewiness, and adhesiveness of composite emulsion gels were increased with the increase of treatment pressure and time. The elasticity reflects the structural damage extent of the samples after initial compression. A relatively low elasticity indicates a serious damage occurred in emulsion gels during compression. The cohesiveness was used to characterize the difficulty to destroy the gel structure of samples. The more compact the gel structure is, the higher the cohesion (35, 36). Compared with other samples, the emulsion gels under the treatment of 400 MPa had a lower elasticity but an extremely higher cohesiveness, which might be explained by the fact that 400 MPa is close to the critical pressure of gel formation. Although the sample treated under 400 MPa also exhibited a gel-like property, the regularity of the gel network and the strength of interaction between the networks were very weak, thereby decreasing the gel strength of the sample.

As the WPI concentration was increased, the gel properties of the samples were enhanced. Especially, the growth in cohesiveness was slowed down, accompanied by a reduced elasticity as well as the occurrence of broken phenomenon during the secondary chewiness. On the other hand, the increase in the κ-CG concentration also led to the increase in gel strength. As reported by Schmitt and Turgeon (37), highly negative-charged polysaccharide can increase the hardness of a gel, but also making them more brittle and difficult to recover after shearing. However, the recovery ability of a gel is dominated by the interaction strength between protein and polysaccharide. The emulsion gel samples containing 10% (w/v) WPI and 1% (w/v) κ-CG have the same WPI/κ-CG ratio with samples containing 12% (w/v) WPI and 1.2% (w/v) κ-CG; however, only the latter one showed a decreased gel strength. This phenomenon suggested that although WPI and κ-CG had a synergistic effect on the formation of composite emulsion gels, an excessively high WPI/κ-CG concentration would lead to the formation of an excessively compact gel structure, decreasing their protective effects against external force.

### Fluorescence Spectral Analysis

The emulsions with a WPI and κ-CG concentration of 20 wt% (w/w) and 2 wt% (w/w) were prepared with the same method described above prior to treating with different pressure and time. The samples were in a liquid state since the concentration of WPI and κ-CG were not high enough to form a gel structure. The samples were performed a fluorescence spectral analysis in order to evaluate the structure change of WPI in emulsions during UHP treatment.

It is well-known that the endogenous fluorescence of protein is mainly ascribed to the tryptophan (Trp), tyrosine (Tyr), and phenylalanine (Phe) in protein, with a fluorescence intensity ratio of 100:9:0.5. This signifies that the fluorescent character of protein containing Trp mainly depends on the existence of Trp. β-lactoglobulin, the main component of WPI, is able to fluoresce under excitation mostly due to the hidden Trp19, rather than Trp61 at the surface, while the fluorescence of α-lactalbumin derives from its four Trp residues. The maximum emission wavelength (λ_max_) of the emulsion samples was about 328 nm, which is between 320 and 350 nm, the maximum emission range of Trp.

**Figure 4:**
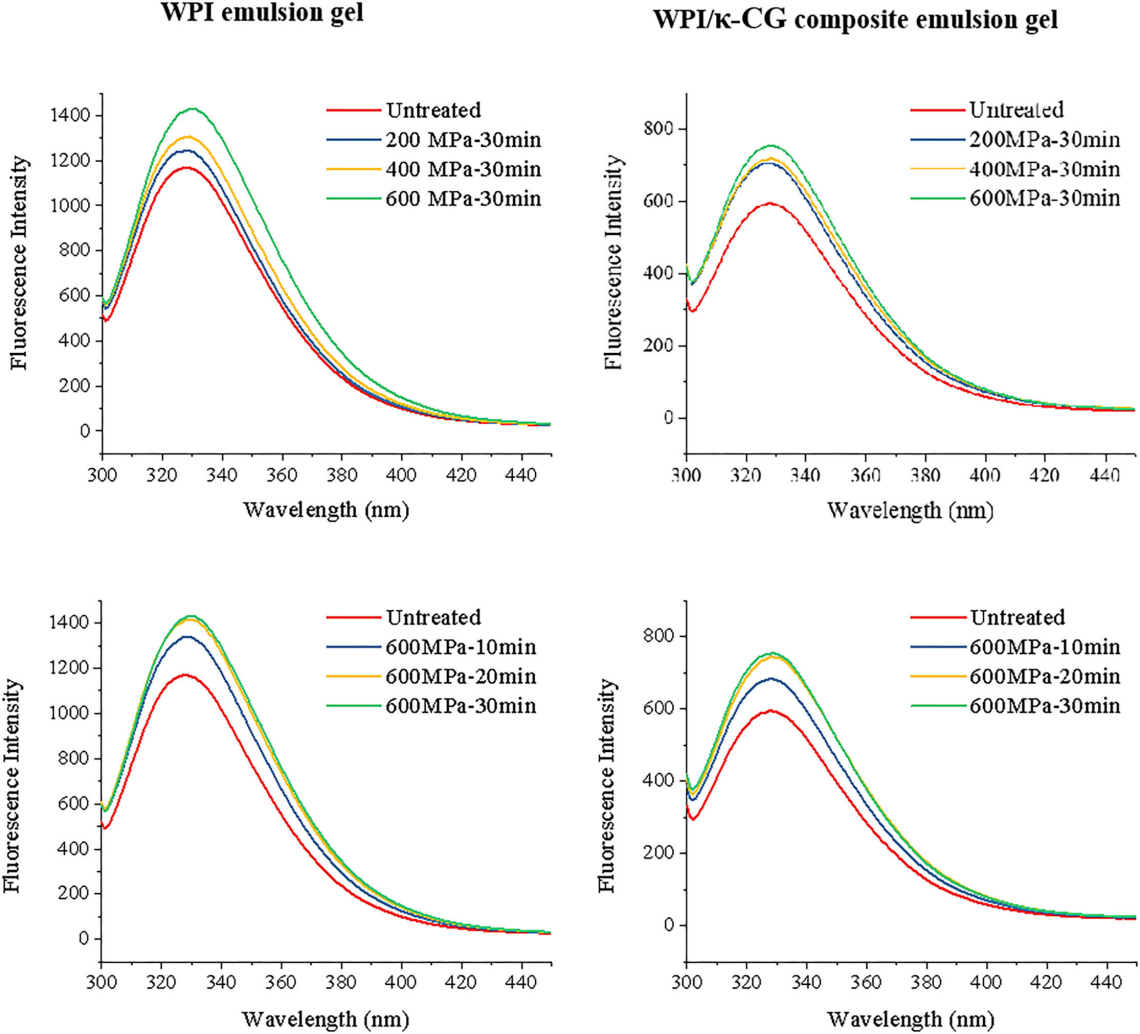
Fluorescence spectra of WPI and WPI/κ-CG composite emulsions with different processing conditions.

The high-pressure treatment will denature the protein molecules, and the microenvironment of fluorescent amino acid groups can be transferred from the polar hydrophilic surface to the hydrophobic interior, thereby increasing the fluorescence intensity of WPI and WPI/κ-CG in emulsions. As shown in Figure 4, the fluorescence intensity was increased with the increasing processing pressures (0.1~600 MPa) and time (0~40 min). The fluorescence intensity was obviously increased when the pressure was higher than 200 MPa, which indicated that the WPI was denatured at this moment. Either WPI emulsions or WPI/κ-CG composite emulsions, the fluorescence intensity curve of the sample treated at 600 MPa for 20 and 30 min was nearly coincident. Presumably, the WPI molecules had cross-linked with each other and formed a relatively stable intermolecular structure under this condition, while the molecular structure of WPI was further destroyed by UHP treatment, thereby leading to an enhancement in fluorescence. The fluorescence intensity (I_f_) of the untreated WPI/κ-CG emulsion was about 594.98, which was much lower than that of the WPI emulsion (1168.38), corresponding to a λ_max_ value of 328 nm. The phenomenon is so-called quenching of protein. The quenching would be ascribed to the Trp, Tyr, or Phe in protein, which were covered up during the binding process of nonfluorescent molecular κ-CG with WPI, preventing the exposure of these chromophore. Similar phenomenon was also reported in a previous research (38), where κ-CG could modify the tertiary structure of peanut protein, as well as adsorb onto the surface of peanut protein, which prevents the exposure of Trp to the aqueous phase.

### Effect of UHP on Secondary Structure of Protein

Table 4 shows the secondary structure of WPI in WPI and WPI/κ-CG solution with or without UHP treatment, calculated by DICHROWEB. The results indicated that the α-coil content was significant decreased, while the β-sheet, β-turn, and random-coil contents were increased (among them, the content of β-sheet increased most significantly) when WPI and κ-CG were combined under atmospheric pressure. Considering the fact that κ-CG is not able to induce the damage in the secondary structure of WPI molecules under normal pressure, it can be deduced that the interaction between κ-CG and WPI promoted the production of intermolecular β-sheets between WPI molecules. The intramolecular and intermolecular hydrogen bonds are responsible for the α-helix and β-sheet of protein, respectively, which are strongly influenced by hydrophobic and electrostatic interactions (39–41). Li et al. (42) have proposed that the higher electrostatic repulsion between protein molecules may be the reason for the decrease of random coil content and the increase of α-helix content in secondary structure. Therefore, the change of secondary structure content in this experiment was mostly due to the fact that the electrostatic interaction between WPI and κ-CG reduced the electrostatic repulsion between WPI molecules. Examples of polysaccharides affecting the secondary structures of proteins are abundant and the effect of polysaccharides on the secondary structure of proteins was verified to be related to the types of proteins and polysaccharides. For example, the acacia gum was found to be able to cause about 70% loss of α-helix in β-lactoglobulin (43), and pectin was also found to change the α-helix content of β-lactoglobulin (44).

**Table 4:** Changes in secondary structure of WPI and WPI/κ-CG complexes.

**Sample**	**Pressure (MPa)**	**Time (min)**	**α-coil (%)**	**β-sheet (%)**	**β-turn (%)**	**Random-coil (%)**
WPI	0.1	0	19.1	29.6	21.1	30.3
	200	30	18.5	29.0	21.6	30.9
	400	30	19.6	27.3	21.8	31.4
	600	10	18.0	28.1	22.1	31.9
		20	17.8	27.9	22.2	32.1
		30	17.1	28.1	22.2	32.2
WPI/κ-CG	0.1	0	11.6	34.7	21.9	31.8
	200	30	11.7	34.6	21.9	31.9
	400	30	10.7	34.7	22.3	32.4
	600	10	11.5	34.3	22.1	32.1
		20	10.3	34.9	22.3	32.6
		30	10.7	34.7	22.3	32.4

The α-helix and β-sheet content of WPI in WPI emulsion was decreased at a relatively low pressure of 200 MPa but increased at 400 MPa. This was mostly due to the fact that the secondary structure of WPI was unfolded at a low pressure, while the increased pressure facilitated the refolding of protein molecules and the intermolecular interactions between protein molecules, which jointly contributed to the formation of the gel network. This phenomenon was consistent with a previous report by Acero-Lopez et al. (45) who have reported that the treatment at 200 MPa converted the secondary structure of ovotransferrin from α-helix, β-sheet, β-turn, and aggregate chains into intermolecular β-sheet or aggregated strands, leading to the rearrangement of ovotransferrin under high-pressure conditions. With the UHP treatment under 600 MPa, the content of α-helix and β-sheet was decreased, which could be explained by the fact that the high pressure damaged the gel network in emulsions. Moreover, the β-turn and random coil were increased with the increase of the processing pressure and time, which was agreed with a previous research of Maria et al. (46).

Compared with WPI emulsions, the WPI/κ-CG composite emulsions showed a relatively small change, and the change tended to occur under relatively high pressure. For example, the decrease of α-helix and β-sheet was found until the pressure was increased to 400 MPa. Besides, under the treatment of 600 MPa, the α-helix and β-sheet contents showed a trend of decrease-increase and decrease-increase-decrease, respectively, with the increase of treatment time. This phenomenon could be ascribed to the fact that the intermolecular interactions existed in the WPI/κ-CG composite emulsions are not liable to be destroyed by the UHP treatment since the κ-CG molecules were beneficial to protect the secondary structure of WPI.

### Fourier Transform Infrared Spectroscopy

**Figure 5:**
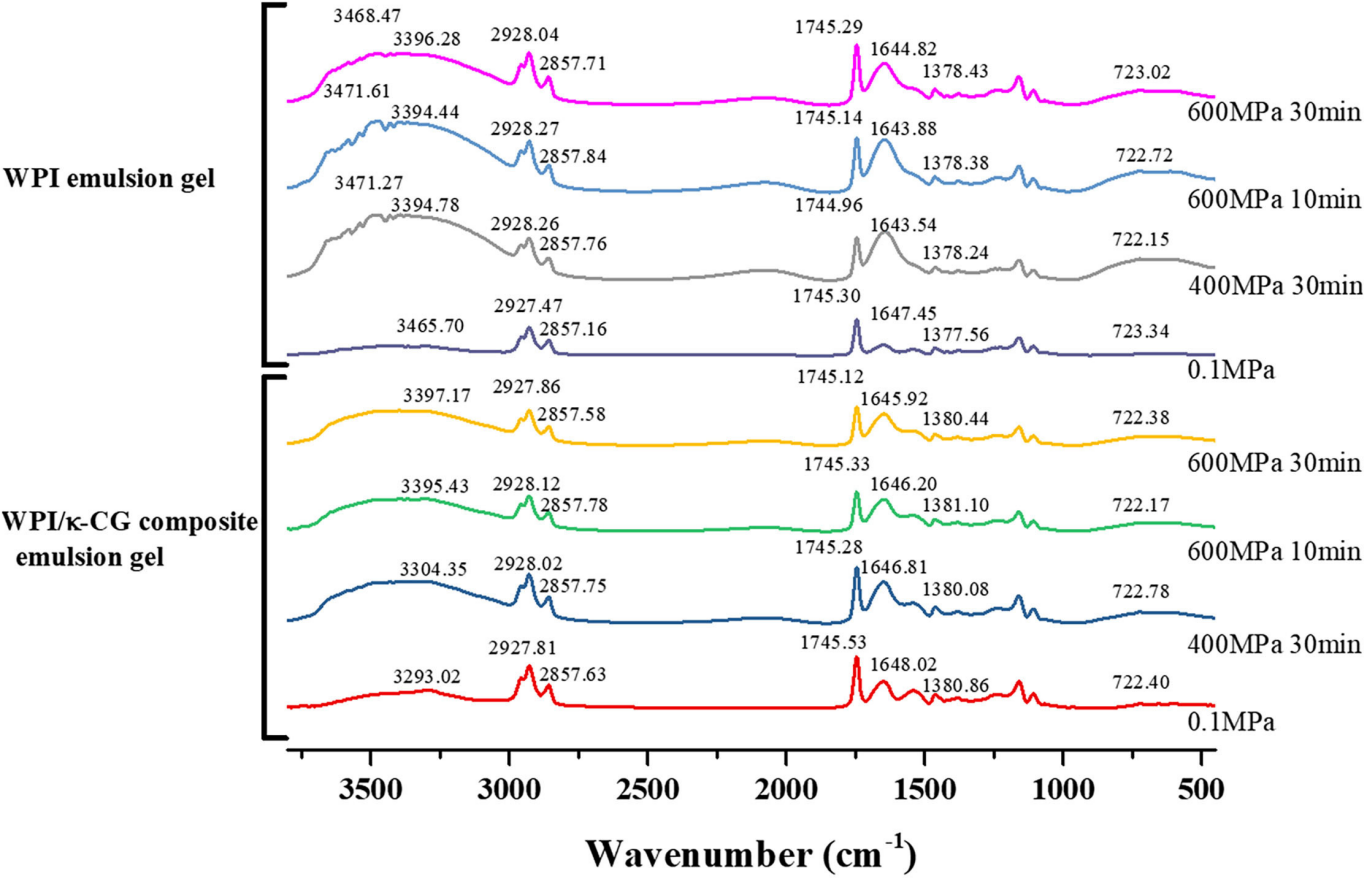
Fourier transform infrared spectroscopy (FTIR) spectra of samples treated with different pressure and time.

The FTIR spectroscopy is often used to analyze the composition of functional groups and the conformational changes in biomacromolecules. The conformation and flexibility of biomacromolecules, such as proteins and polysaccharides, are responsible for their intermolecular interactions, chemical properties, and functional properties (47). Figure 5 depicts the FTIR spectra of WPI and WPI/κ-CG composite emulsions with or without the UHP treatment under different processing pressure and time. For both WPI and WPI/κ-CG composite emulsion gel samples, as the treatment pressure or time increases, a red shift could be seen in the broad absorption peaks in 3,200~3,500 cm^−1^ region (amide A band), which is the characteristic for the intermolecular vibration of hydrogen bond O-H stretching. Taking WPI emulsion gels as an example, the absorption peak of the sample without high-pressure treatment was at the wave number of 3293.02 cm^−1^, which was shifted to a higher wave numbers at 3304.35 cm^−1^, 3394.43 cm^−1^, and 3397.17 cm^−1^ after UHP treatment under 400 MPa for 30 min, 600 MPa for 10 min, and 600 MPa for 30 min, respectively. Therefore, it could be inferred that the UHP treatment could impact the hydrogen bonding of WPI molecules, weakening their affinity to water molecules, as well as enhancing the interactions between WPI molecules. Compared with WPI emulsion gels, a red shift in the corresponding absorption peak of WPI/κ-CG composite gels could be also observed, which was mostly due to the hydroxyl vibration of κ-CG (44). Moreover, the sharper absorption peak characterizing N-H stretching vibration gradually occurred in the spectra of WPI/κ-CG composite gels near the wave number of 3,470 cm^−1^, which would be blue shifted when the N-H group of the WPI participated in the formation of hydrogen bond in an α chain.

It could be observed that the untreated emulsion sample had a peak at the wavenumber of 1542.27 cm^−1^, which was associated with the stretching vibration of C–N group and bending vibration of N–H group (amide II). After treated by UHP, a decrease in the intensity of the absorption peak for amide II occurred. At the same time, the peak at 1648.02 cm^−1^, which was the characteristic peak of amide I (C=O stretching vibration and C-N bending vibration), was red shifted with a significantly increased intensity. These phenomena suggested that an increase in the coiled structure of emulsion gel samples, in other words, a decrease in the ordered structure, due to the formation of WPI/κ-CG complex (48, 49). According to the report of Timilsena et al. (50) negatively charged sulfate groups in κ-CG were able to combine with the positively charged amide groups in polypeptide chains to form polyelectrolyte complexes, leading to higher amplitudes in amide I. Therefore, it could be deduced that the κ-CG and WPI molecules in composite emulsions were combined with each other, exerting a synergistic effect on the formation of the gel structure. As the processing pressure and time increases, the absorption peak near 1542.27 cm^−1^ in the amide II region was red shifted, which might be due to the fact that the hydrophilic groups containing in κ-CG molecules could affect the intermolecular hydrogen bond between WPI molecules.

### Microstructure of Emulsion Gels

**Figure 6:**
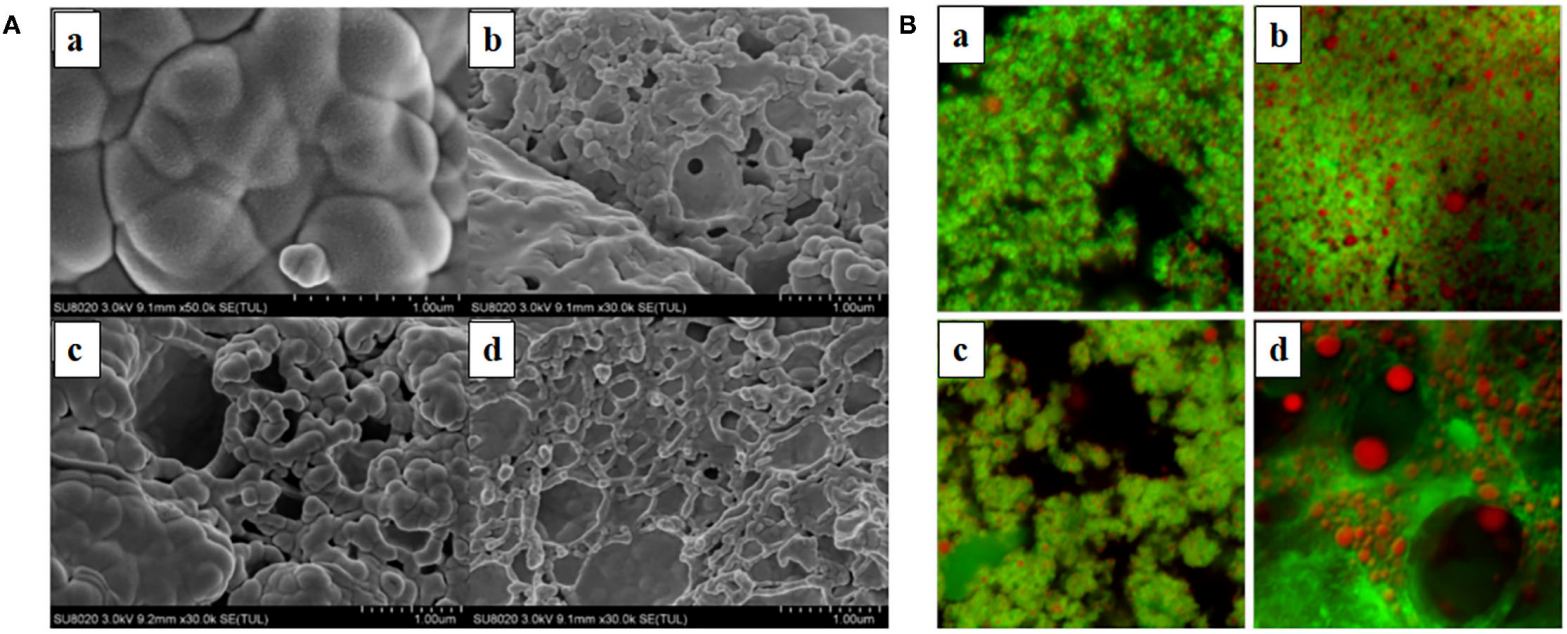
Emission scanning electron microscope (SEM) **(A)** and confocal laser scanning microscopy (CLSM) **(B)** images of emulsion gels treated with different pressure and time. The MCT concentration was 20% (w/w), and the aqueous phase contained 10% (w/v) WPI and 1% (w/v) κ-CG. a–d were the images of untreated and UHP-treated emulsion gel samples under 400 MPa for 30 min, 600 MPa for 10 min, and 600 MPa for 30 min, respectively.

The SEM images of emulsion gels were captured using SEM to characterize their microstructure. As depicted in Figure 6, for samples without UHP treatment, nanosized particles were suspended and aggregated in emulsions, forming large particle aggregations. However, after UHP treatment, clear filament-like cross-linked structure or spherical aggregate with large cavity was formed as the processing pressure and time increase. It could be also observed that the greater the intermolecular bond and the larger the reactivity of protein side chains, the more regular the connection of the WPI molecules, in agreement with several previous reports (51, 52). Previous experiments have confirmed that the emulsion gel formed under 400 MPa was softer, moister, and more liquid-like than the emulsion gel formed under 600 MPa, which could be also explained by the microstructure of these emulsion gels. The regular three-dimensional gel network could provide a good support for the gel structures in emulsions, endowing them with an enhanced hardness and elastic properties. A large amount of moisture was blocked within the pore formed by WPI, hindering the freely flow of water molecules.

For composite gels containing κ-CG, only a low concentration of WPI was required to form an emulsion gel with relatively high hardness with the UHP treatment under 600 MPa for 30 min, suggesting that the electrostatic interaction between κ-CG and WPI was beneficial to the formation of gel structure in emulsions. It could be also found that the gel network of WPI/κ-CG composite emulsion was loose and disordered with large and uneven cavities. This might be due to the fact that the gel structure in emulsion gels induced by UHP treatment depended on the protein unfolding and aggregation rate. The introduction of κ-CG molecules enhanced the viscosity of emulsions, hindering the movement of protein molecules and the aggregation of WPI molecules (53). Another possible explanation might be that the WPI concentration employed in the composite emulsion gel was not sufficient to form a dense network structure.

Figure 6B shows the CLSM image of samples with a magnification of 100x. The MCT was stained with Nile Red (presenting in blue), whereas the WPI was stained by Nile Blue (presenting in red). The size of oil droplets was increased as the processing pressure increases, which indicated that the UHP treatment contributed to the aggregation and coalescence of oil droplets. When the processing pressure was higher than 400 MPa, the oil droplets were embedded within a regular network composed of WPI molecules. Moreover, unlike the emulsion gel treated under 400 MPa for 30 min, whose gel network was indistinct, the emulsion gel treated under 600 MPa for 30 min exhibited a clear structure with obvious boundary. However, the droplet size was not uniform and large cavities could be seen, further confirming the results obtained by SEM observation.

### Intermolecular Force of Emulsion Gels Induced by UHP

**Figure 7:**
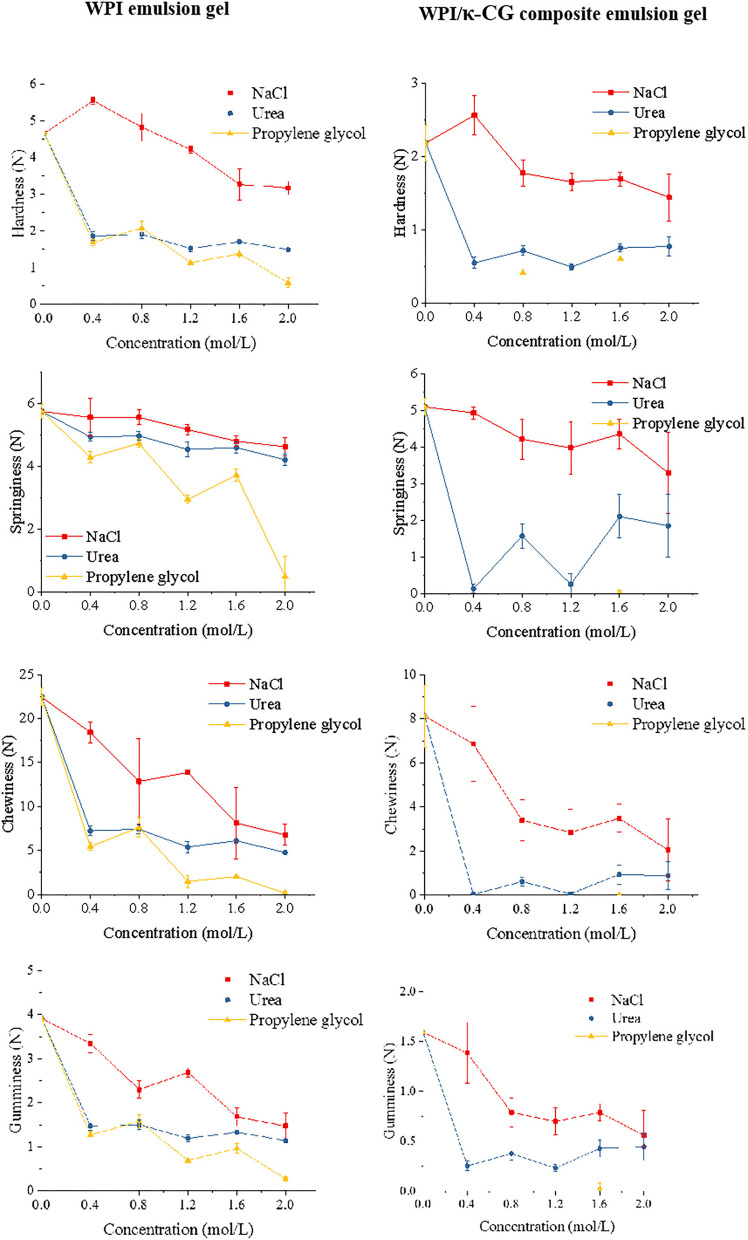
Effect of different concentrations of NaCl, urea, and propylene glycol on the texture properties of emulsion gels.

In this experiment, three kinds of bond cleavage agents, NaCl, urea, and propylene glycol were used to destroy the main non-covalent bonds in emulsion gels. These bond cleavage agents are able to cause the irreversible molecular rearrangement of WPI, which was manifested as the texture changes in emulsion gel samples. The addition of NaCl into emulsion gels introduced a large amount of Na^+^, which could adsorb onto the surface of protein molecules and lead to an electrical shielding effect, thereby reducing the electrostatic interactions between protein molecules. Urea was primarily used to destroy the hydrogen bonds in emulsion gel samples, replacing protein-protein and protein-water interactions through forming more stable protein-urea hydrogen bonds; in this case, more hydrophobic residues were exposed, preventing the formation of gel networks (54). The propylene glycol was used to inhibit the hydrophobic interaction in emulsion gels. Since the materials utilized in the current work (WPI and κ-CG) were biological macromolecules, the Van der Waals force could be ignored (too weak). Although the SD of the data in this section was relatively large due to the inevitable systematic errors (e.g., slight thickness difference or sample tilting when the gel was too soft), significant changes could be found in the texture of emulsion gels, as shown in Figure 7.

The existence of a low level of ion can effectively weak the intermolecular repulsion through combining with amino acids and water molecules. As the NaCl level increases, the strength of emulsion gels was remarkably increased. However, an extremely high NaCl level led to the reduction in the attractive forces as well as the increase in the repulsion or dissociation between protein molecules, thereby weakening the cross-linking between protein chains (55). As shown in Figure 7, NaCl with a concentration of 0.4 mol/L contributed to increase the hardness of emulsion gels, whereas the hardness was significantly decreased as the NaCl concentration was further increased. With the addition of urea, the WPI emulsion gel could remain in its texture. It could be also observed that the elastic was nearly unchanged in WPI emulsion gel, but changed obviously in WPI/κ-CG composite emulsion gel. This phenomenon indicated that κ-CG promoted the formation of intermolecular hydrogen bonds, which led to the destruction of gel structure during the initial compression process in the TPA measurement mode. Noteworthily, the effect of propylene glycol on the texture of emulsion gel was most obvious, indicating that the hydrophobic interaction is the critical factor that affected the formation of these emulsion gels. Moreover, the WPI/κ-CG composite gel treated with propylene glycol was excessively soft, even not achieving the requirement for a TPA measurement, indicating that the hydrophobic interaction is the most important factor that affects the formation of the gel structure in composite gels.

### *In vitro* Gastrointestinal Digestion

**Figure 8:**
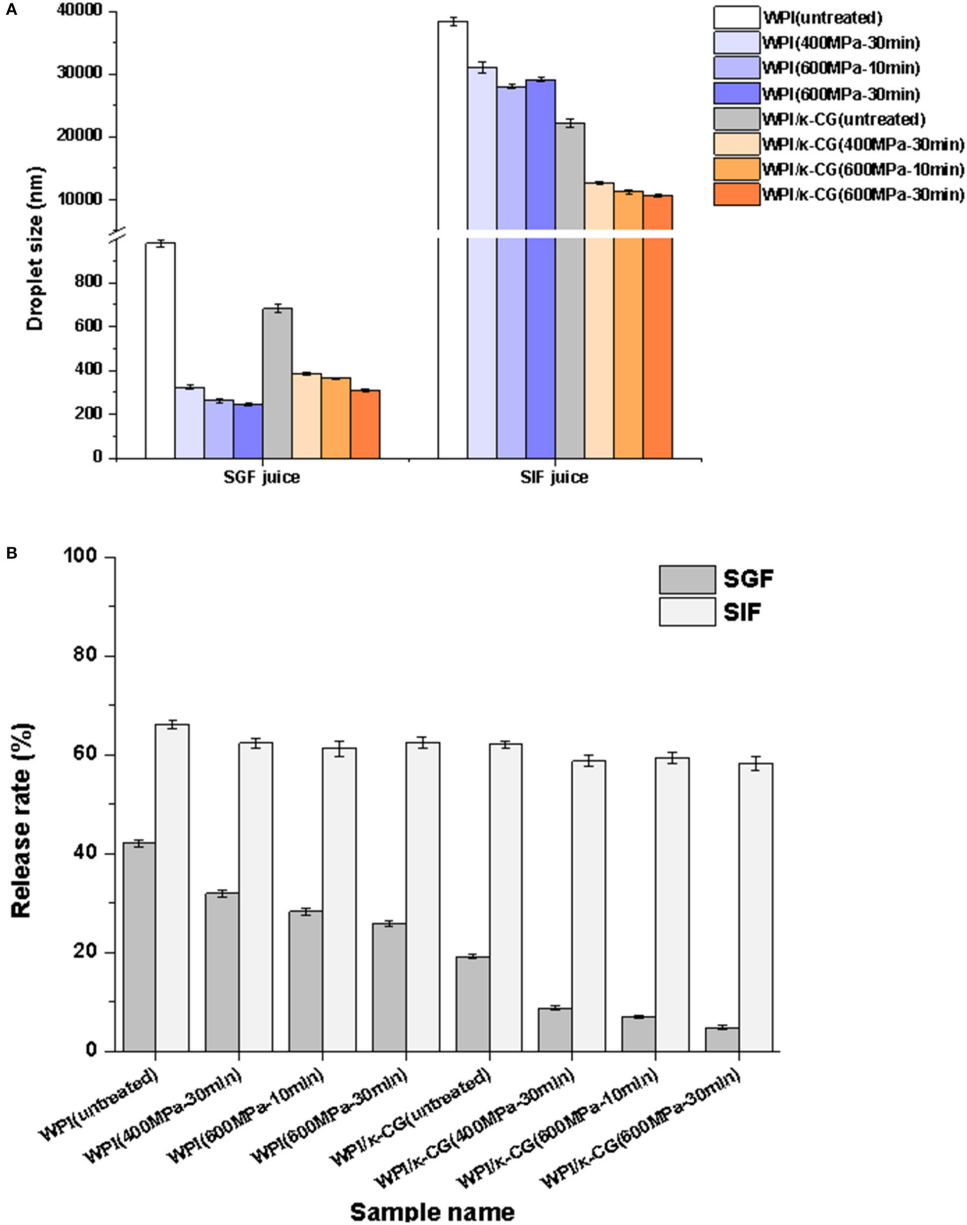
**(A,B)** Droplet size after simulated gastric fluid (SGF) and simulated intestinal fluid (SIF) digestion and bioacessibility of curcumin of WPI and WPI/κ-CG composite emulsion gels with different treatments.

Figure 8A presents the changes in the droplet size of WPI and WPI/κ-CG emulsion gels after SGF and SIF digestion. Apparently, whether for WPI or WPI/κ-CG emulsion gels, the UHP treatment samples exhibited a significant decrease in the droplet size compared to that of untreated samples after SGF and SIF digestion. It could be also observed that there was also a significant difference in the particle size of emulsion gels with different processing conditions. For the same sample, the increase in both treatment time and pressure led to a decrease in the droplet size of SGF and SIF digestion juice. One of possible explanations might be that the UHP treatment led to a more compact structure in these emulsion gels, which would further inhibit the digestion. Moreover, the droplet size of WPI emulsion gels was significantly smaller than that of WPI-κ-CG composite emulsion gels after SGF digestion. On the contrary, the WPI emulsion gels were determined with larger droplet size compared with that of WPI-κ-CG composite emulsion gels after SIF digestion. This result might be attributed to the difference in the droplet size of the untreated WPI emulsion and WPI-κ-CG composite emulsion.

The lipid would be hydrolyzed into diacylglycerols and monoglycerols during digestion, leading to the release of free fatty acid (FFA). The released FFA at the oil/water interface is liable to combine with bile salts and phospholipids to form micelles. The curcumin in micelles is considered to be solubilized and bioaccessible. As presented in Figure 8B, the cumulative release of curcumin in UHP-treated emulsion gels was slightly decreased compared to that of untreated emulsions. Moreover, the treatment pressure and time had nearly no effect on the cumulative release of curcumin in emulsion gels during *in vitro* simulated digestion. Interesting enough, the incorporation of κ-CG was confirmed to contribute to the controlled release of curcumin. Most of the curcumin encapsulated within WPI/κ-CG composite emulsion gels was released in SIF stage, whereas the majority of curcumin was released during SGF stage in WPI emulsion gels.

## Conclusions

In this work, the WPI/κ-CG composite emulsion gels were successfully prepared through UHP treatment. The structure of the emulsion gels was mainly maintained by hydrophobic interaction and hydrogen bonding. The UHP treatment can lead to the denaturation of WPI in the emulsion gels. As the pressure increases, the secondary structure of WPI undergoes a process of destruction-forming, and the UHP treatment under a longer time can cause a secondary destruction. Moreover, κ-CG was able to combine with WPI through electrostatic interaction, protecting the WPI molecules against denaturation to a certain extent. The results also confirmed that the UHP treatment significantly retarded the release of curcumin but was beneficial to the controlled release of curcumin. These findings not only developed a non-thermal process to fabricate emulsion gels, but also provided useful information for the development of protein/polysaccharide composite emulsion gels as effective delivery systems for the encapsulation of thermal sensitive functional components.

## Data Availability Statement

The original contributions presented in the study are included in the article/supplementary material, further inquiries can be directed to the corresponding author/s.

## Author Contributions

JS: conceptualization, methodology, and writing-original draft preparation. LW and XL: visualization and investigation. WD: data curation. JW: software. JY: visualization. FR: supervision. FY and PW: writing-reviewing and editing. All authors contributed to the article and approved the submitted version.

## Funding

This research was funded by the National Natural Science Foundation of China (No. 31901625) and the National Natural Science Foundation of China (No. 31371836).

## Conflict of Interest

The authors declare that the research was conducted in the absence of any commercial or financial relationships that could be construed as a potential conflict of interest.

## Publisher's Note

All claims expressed in this article are solely those of the authors and do not necessarily represent those of their affiliated organizations, or those of the publisher, the editors and the reviewers. Any product that may be evaluated in this article, or claim that may be made by its manufacturer, is not guaranteed or endorsed by the publisher.
